# A Highly Durable Rubber‐Derived Lithium‐Conducting Elastomer for Lithium Metal Batteries

**DOI:** 10.1002/advs.202200553

**Published:** 2022-03-31

**Authors:** Yongzheng Shi, Na Yang, Jin Niu, Shubin Yang, Feng Wang

**Affiliations:** ^1^ State Key Laboratory of Chemical Resource Engineering Beijing Key Laboratory of Electrochemical Process and Technology for Materials Beijing University of Chemical Technology Beijing 100029 P. R. China; ^2^ Beijing Advanced Innovation Center for Soft Matter Science and Engineering Beijing University of Chemical Technology Beijing 100029 P. R. China; ^3^ School of Materials Science and Engineering Beihang University Beijing 100191 P. R. China

**Keywords:** ionic conductivity, lithium metal batteries, rubber‐derived elastomer, solid polymer electrolyte, vulcanization approach

## Abstract

Elastomers offer attractive advantages over classical solid‐state electrolytes in terms of ensuring stable interfacial contact and maintaining fatigue durability, but the low ionic conductivity obstructs their practical applications in long‐life lithium metal batteries. In this work, rubber‐derived lithium‐conducting elastomer has been developed via sulfur vulcanization of nitrile butadiene rubber with a polymerizable ionic liquid to provide both high resilience and dramatically improved ionic conductivity. Owing to the chemically crosslinked network between rubber chains and ionic liquid fragments generated during vulcanization, the elastic lithium‐conductor achieves high resilience of 0.92 MJ m^−3^, superior cyclic durability of 1000 cycles at 50% strain, and high room‐temperature ionic conductivity of 2.7 × 10^−4^ S cm^−1^. Consequently, the corresponding solid‐state lithium/LiFePO_4_ battery exhibits a high capacity of ≈146 mAh g^−1^ with a high capacity retention of 94.3% for up to 300 cycles.

## Graphical abstract

By virtue of a chemically crosslinked network via vulcanization approach, the rubber‐derived lithium‐conductor achieves high resilience of 0.92 MJ m^−3^, superior fatigue durability of 1000 cycles at 50% strain, and high room‐temperature ionic conductivity of 2.7 × 10^−4^ S cm^−1^. This unique lithium‐conducting elastomer enables intimate contact and fast lithium‐ion transport, resulting in long‐life and high‐performance solid‐state lithium metal batteries.

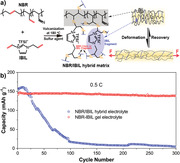

## 1 Introduction

The ever‐increasing demand for energy storage systems with high‐energy density, high safety, and long‐cycling life to power mobile electronics and electric vehicles has shifted the focus from organic liquid electrolyte‐based lithium batteries toward solid‐state batteries.^[^ ^1^, ^2^ ^]^ In order to avoid the use of volatile and thermally unstable liquid electrolytes, solid‐state lithium‐ion conductors with high ionic conductivity, improved electrochemical stability, and good mechanical properties are urgently needed.^[^ ^3^, ^4^ ^]^ Moreover, with the fast development of advanced energy storage systems with high‐energy density, mechanical properties of solid‐state electrolytes are becoming a key factor in withstanding repeated volume changes of electrodes (e.g., metallic lithium anodes, silicon anodes, and sulfur cathodes) and large deformations of pouch cells.^[^ ^5^ ^]^ There are two main types of solid‐state electrolytes: inorganic solid electrolytes and solid polymer electrolytes (SPEs).^[^ ^6^ ^]^ The former include sulfides, oxides, nitrides, and phosphates, and can provide high ionic conductivities (10^–4^–10^–2^ S cm^–1^) close to or even higher than liquid electrolytes, but their brittle nature, high reactivity, and high interfacial impedance with electrodes hinder their practical applications.^[^ ^7^ ^]^ Compared to inorganic solid electrolytes, SPEs consisting of a polymer matrix and lithium salts possess significant processing advantage and superior interfacial contact, as well as comparable safety enhancements.^[^ ^8^ ^]^ Unfortunately, pristine polymer electrolytes with high mechanical strength usually have poor room‐temperature ionic conductivity^[^ ^9^ ^]^ of less than 10^–5^ S cm^–1^. The introduction of small molecule additives can plasticize or gelatinize polymer electrolytes and improve their ionic conductivity but significantly reduce the mechanical strength,^[^ ^10^, ^11^ ^]^ causing irreversible plastic deformation or fracture at small strains and unstable interface contact with electrodes. Therefore, there is an urgent need for SPEs with both high mechanical performance and good ionic conductivity.

Elastomers are an ideal choice for solid electrolytes in long‐life batteries due to their high resilience to accommodate repeated volume changes of advanced electrodes during operation.^[^ ^12^ ^]^ In the case of lithium metal batteries, elastomers can afford stable contact with electrodes even when metallic lithium anodes suffer from relatively infinite volume changes during deep charge/discharge cycling. Moreover, elastomers can be assembled with stretchable electrodes to form multifunctional batteries, thus powering wearable electronic devices such as healthcare devices, stretchable sensors, and implantable devices.^[^ ^13^ ^]^ To date, only a few lithium‐conducting elastomers with high ionic conductivities have been reported. For example, elastic poly‐butyl acrylate/succinonitrile^[^ ^14^ ^]^ (PBA/SN) and SiO_2_/poly(propylene oxide)^[^ ^15^ ^]^ (PPO) electrolytes were fabricated via polymerization‐induced phase separation and covalent‐dynamic hydrogen bonding dual‐crosslinking method, respectively, and the corresponding solid‐state lithium metal batteries operated well at room temperature. However, most reported elastomer‐based electrolytes are either poor at lithium conducting or suffer mechanical deterioration due to gelation. Therefore, new approaches need to be developed toward a resolution of the dilemma between ionic conductivity and mechanical properties.

In this work, a rubber‐derived lithium conductor (denoted NBR/IBIL hybrid electrolyte) has been fabricated by vulcanization of nitrile butadiene rubber (denoted NBR) with a polymerizable ionic liquid (1‐allyl‐3‐vinylimidazolium bis(trifluoromethylsulfonyl)imide denoted IBIL) in an attempt to resolve the above dilemma. The NBR/IBIL hybrid electrolyte inherits the elasticity and fatigue durability of NBR by virtue of a chemically crosslinked network of NBR chains and IBIL fragments generated via free radical addition reaction during vulcanization. The resulting lithium‐conducting elastomer possesses high resilience (0.92 MJ m^–3^) and long‐lasting cyclic durability (1000 cycles) at a high strain of 50%. Furthermore, the elastic NBR/IBIL hybrid electrolyte achieves a high room‐temperature ionic conductivity of 2.7 × 10^–4^ S cm^–1^ as a result of fast lithium‐ion pathways paved by IBIL fragments. Consequently, the NBR/IBIL hybrid electrolyte provides stable contact with the electrodes and fast lithium‐ion transport, resulting in the high performances of solid‐state lithium metal batteries, including long cycling life of 2000 h for the symmetrical battery and 300 cycles for the full battery.

## 2 Results and Discussion

**Figure 1:**
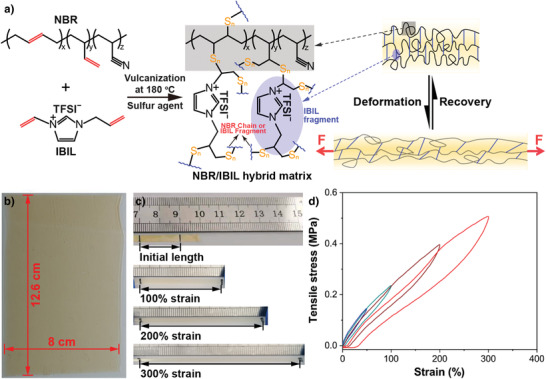
a) Synthesis of the NBR/IBIL hybrid electrolyte with a schematic illustration of the segmental motion of NBR chains and IBIL fragments as crosslinkers in the hybrid electrolyte during deformation and recovery (LiTFSI is omitted). b) Image of the NBR/IBIL hybrid electrolyte on a glass plate. c) Images of the NBR/IBIL hybrid electrolyte under different strains and d) its stress–strain cycling in the strain range from 50% to 300%.

The elastic lithium‐conductor consists of an NBR/IBIL hybrid matrix and a lithium salt (lithium bis(trifluoromethylsulfonyl) imide, denoted LiTFSI). As schematically illustrated in **Figure** **1**a, the NBR/IBIL hybrid matrix is synthesized via vulcanization of NBR and polymerizable IBIL with a vulcanizing agent elemental sulfur at 180 °C, and the detailed synthesis procedures are described in Experimental Section (see Supporting Information). Noted that NBR and vulcanized NBR (denoted v‐NBR) electrolytes were also fabricated in the same procedures except that sulfur/IBIL or IBIL was not added to the precursor solutions (see Supporting Information), respectively. In this vulcanization approach, IBIL fragments generated during vulcanization are chains of IBIL molecules joined by the sulfur agent and can be utilized as crosslinkers for NBR chains, thus avoiding the gelation of NBR electrolytes. Under an applied tensile stress, these IBIL fragments provide mechanical strength and allow the NBR‐based elastomer to recover from deformation, in contrast to gelatinized electrolytes which undergo severe degradation of mechanical properties during deformation. As shown in Figure 1c,d, the NBR/IBIL hybrid electrolyte can be stretched to 300% of its initial length without breaking and recover from various strains, demonstrating its high elasticity and resilience. The hybrid electrolyte membrane has an area larger than 100 cm^2^ with a dry surface (Figure 1b), with a transparent yellowish color characteristic of vulcanization. Furthermore, the fabrication of the NBR/IBIL hybrid can be readily scaled up by increasing the size of the film applicator and glass substrate. The thickness of the as‐prepared electrolyte is ≈55 µm (Figure S2c, Supporting Information), which is much thinner than most reported poly(ethylene oxide) (PEO)‐based electrolyte membranes.^[^ ^16^, ^17^, ^18^ ^]^

The microstructure of NBR/IBIL hybrid electrolyte was first determined by energy dispersive X‐ray spectroscopy. As shown in Figures S1 and S2 (Supporting Information), carbon, fluorine, sulfur, and nitrogen elements are uniformly distributed on the surface and cross section of the hybrid electrolyte, confirming the homogeneous distribution of IBIL and LiTFSI in the electrolyte. Furthermore, no clear diffraction peaks of crystalline materials are observed in the XRD patterns of the NBR/IBIL hybrid electrolyte^[^ ^18^, ^19^ ^]^ (Figure S3, Supporting Information), which confirms the complete consumption of the sulfur agent during vulcanization. Additionally, the hybrid electrolyte displays an amorphousness peak at around 20° with a much lower normalized intensity than that of the NBR electrolyte, indicating that the former has a higher proportion of amorphous regions and easier local segmental motions of the polymer matrix. Furthermore, chemical structure of the NBR/IBIL hybrid matrix was further characterized by Fourier transform infrared spectroscopy (FTIR) and X‐ray photoelectron spectroscopy (XPS). The characteristic peaks of imidazolium ring and TFSI^−^ anion for IBIL can be observed in the FTIR spectrum^[^ ^20^, ^21^ ^]^ (Figure S4, Supporting Information), demonstrating that IBIL has been incorporated into the hybrid matrix. The –C═C– peak intensity of the NBR/IBIL hybrid matrix is significantly lower than those of NBR and IBIL, which can be attributed to the consumption of –C═C– during the vulcanization process. Moreover, XPS results verify the presence of —C—S— bond in the hybrid matrix (Figure S5, Supporting Information), which proves the generation of the chemically crosslinked network during vulcanization. The thermal endurance of the NBR/IBIL hybrid electrolyte was also evaluated by thermogravimetric analysis (Figure S6, Supporting Information). Only a mass drop (≈2.8 wt%) can be observed at 300 °C for the hybrid electrolyte, which is much lower than those of NBR electrolyte (≈7.6 wt%) and most solid composite electrolytes,^[^ ^22^, ^23^, ^24^ ^]^ and demonstrates that the vulcanization approach is effective in enhancing the thermal stability of NBR‐based elastomers.

**Figure 2:**
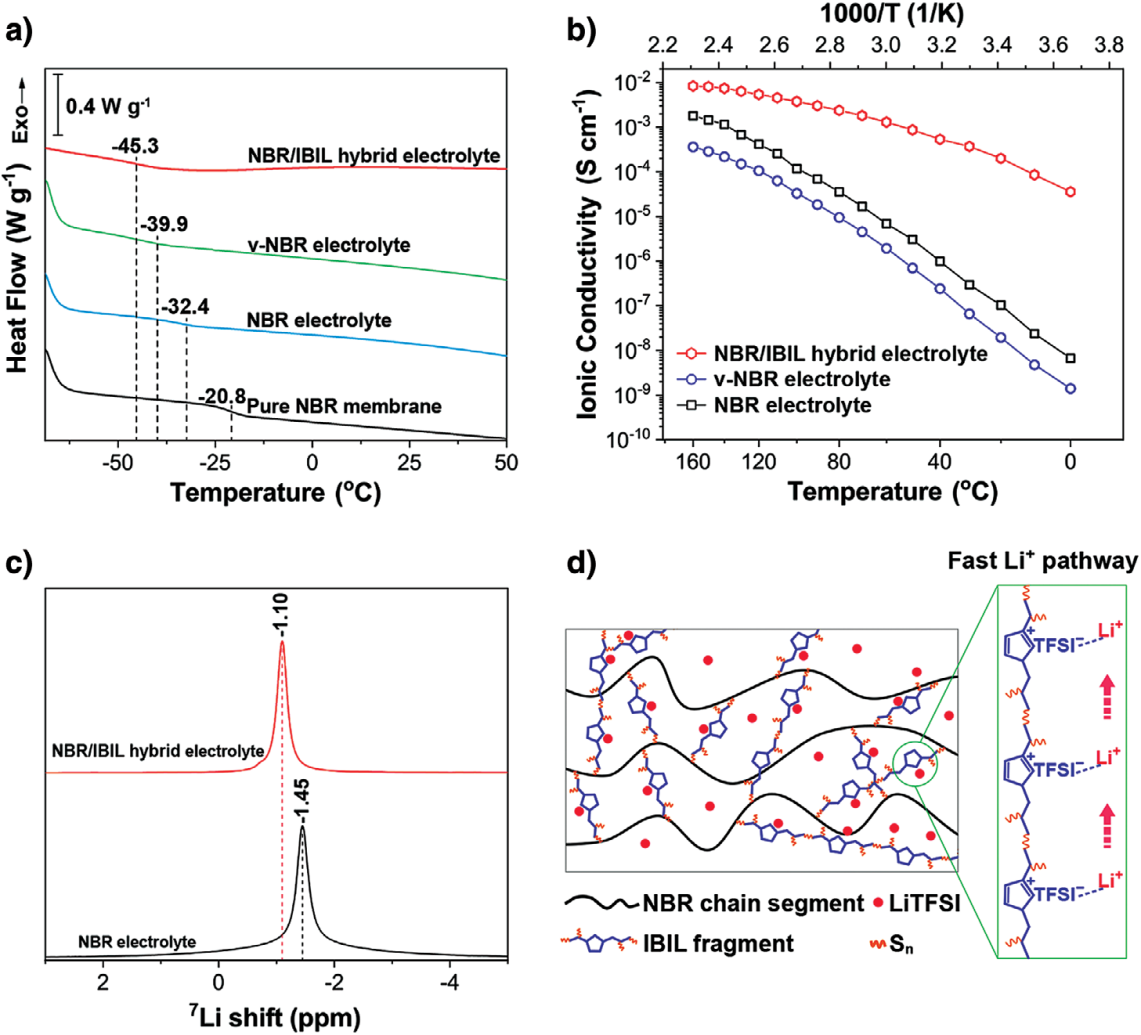
a) Differential scanning calorimetry of the pure NBR membrane and NBR‐based electrolytes. b) Ionic conductivities of NBR‐based electrolytes at varying temperatures in the range 0–160 °C. c) ^7^Li NMR spectra of NBR and NBR/IBIL hybrid electrolytes. d) Schematic illustration of the fast lithium‐ion pathway for NBR/IBIL hybrid electrolyte.

As shown in **Figure** **2**a, the NBR/IBIL hybrid electrolyte has a single glass transition temperature (*T*_g_ = −45.3 °C), which is lower than the other NBR‐based electrolytes, suggesting that the introduction of IBIL fragments has resulted in a high degree of amorphousness and high local mobility of the polymer chain segments at room temperature.^[^ ^25^ ^]^ The width of the *T*_g_ peak is less than 15 K, which is consistent with the random vulcanization of NBR and IBIL and the absence of nano‐ or micro‐phase segregation. To investigate the lithium‐conducting mechanism of the NBR/IBIL hybrid electrolyte, temperature‐dependent conductivities were measured and compared with those of NBR and v‐NBR electrolytes (Figure 2b). All the electrolytes display typical non‐linear Arrhenius plots of ionic conductivity over the temperature range from 0 to 160 °C, and their ionic conductivities increase with increasing temperature, which shows that ion solvation and transport are driven by polymer chain segments and that NBR/IBIL hybrid electrolyte is an SPE. The temperature‐dependent ionic conductivities of the NBR/IBIL hybrid electrolyte can be well‐fitted (Figure S7, Supporting Information) by the Vogel–Tammann–Fulcher equation, further proving its lithium‐ion transport behavior as solid‐state electrolytes. In order to obtain optimized elastic hybrid electrolytes with high ionic conductivity, the contents of IBIL and LiTFSI and curing time were varied. These factors all have a large influence on the ionic conductivity of the NBR/IBIL hybrid electrolyte (Figure S8a–c, Supporting Information). The maximum ionic conductivity of 2.7 × 10^–4^ S cm^−1^ at 25 °C was achieved using 4.8 mmol of IBIL, 1.7 mmol of LiTFSI per gram of NBR with a curing time of 1 h. This value is about three orders of magnitude higher than that of the NBR electrolyte (9.5 × 10^–8^ S cm^–1^), one order of magnitude higher than that of typical PEO‐based composite electrolytes (≈10^–5^ S cm^–1^),^[^ ^26^, ^27^ ^]^ and comparable to that of previously reported elastic SiO_2_/PPO electrolyte.^[^ ^15^ ^]^ Additionally, NBR/IBIL hybrid electrolyte has a low Li‐ion transference number of 0.22 (Figure S9, Supporting Information), which can be attributed to the non‐immobilization of the anion.

The lithium‐conducting mechanism of the NBR/IBIL hybrid electrolyte was further investigated by solid‐state ^7^Li nuclear magnetic resonance (NMR) spectroscopy. The ^7^Li NMR peak of the hybrid electrolyte (Figure 2c) is shifted downfield by 0.35 ppm compared to the NBR electrolyte. This downfield shift indicates a reduced electron density around lithium atoms,^[^ ^28^ ^]^ attributed to looser coordination with the highly electron‐donating groups in the IBIL fragments. Furthermore, the half‐width of the ^7^Li peak is reduced from 0.26 in the NBR electrolyte to 0.20 ppm in NBR/IBIL hybrid, indicating significantly enhanced lithium‐ion mobility in the IBIL fragments.^[^ ^28^ ^]^ Accordingly, IBIL fragments can be deduced to play a considerable role in constructing the lithium‐conducting environment. A possible fast lithium‐ion pathway along IBIL fragments in the NBR/IBIL hybrid electrolyte is schematically represented in Figure 2d. Linear sweep voltammetry measurements (Figure S10, Supporting Information) show that the electrochemical stability window of the hybrid electrolyte is ≈4.6 V versus Li/Li^+^, which is much wider than those of the NBR and v‐NBR electrolytes and comparable to the value for previously reported SiO_2_/PPO electrolyte. Therefore, we can conclude that the as‐prepared NBR/IBIL hybrid electrolyte is a dry membrane with high thermal/electrochemical stability and enhanced ionic conductivity, rather than a gelatinous mixture composed of NBR chains and IBIL molecules.

**Figure 3:**
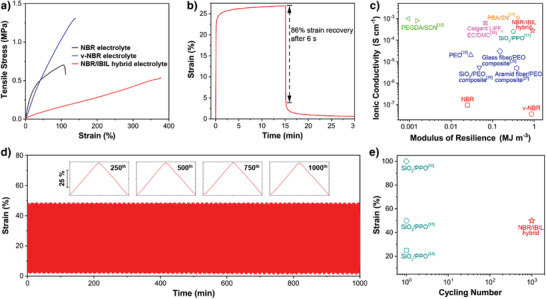
a) Stress–strain curves and b) creep test of NBR‐based electrolytes. c) Plot of room‐temperature ionic conductivities versus modulus of resilience for our NBR‐based electrolytes and SPEs in the literature.^[^ ^10^, ^14^, ^15^, ^26^, ^27^, ^29^, ^30^ ^]^ d) Cyclic durability of NBR/IBIL hybrid electrolyte at 50% strain for 1000 cycles. e) Plot of strain against cycle number for the hybrid electrolyte and SPEs^[^ ^15^ ^]^ in the literature.

The NBR/IBIL hybrid electrolyte shows a rubber‐like mechanical behavior with high resilience and long durability. As shown in **Figure** **3**a, the hybrid electrolyte exhibits a maximum strain of ≈378% at the cost of a slightly lower tensile stress (0.54 MPa) than that of the original NBR electrolyte (0.70 MPa). Thanks to the chemically crosslinked network, the elastic NBR/IBIL hybrid electrolyte presents a linear stress–strain curve similar to v‐NBR and unlike NBR, indexed to little plastic deformation until failure, with its elongation at break being ≈3 times that of NBR and v‐NBR. Additionally, the hybrid electrolyte shows superior resilience to the classical PEO electrolyte and even the pure NBR membrane (Figure S11, Supporting Information). It is noted that as the curing time increases, the tensile stresses of hybrid electrolytes at break increase rapidly (Figure S12, Supporting Information), while uncured gelatinous electrolyte (denoted NBR‐IBIL gel electrolyte, Supporting Information) with the same composition as the NBR/IBIL hybrid electrolyte tends to fracture upon small deformations due to the absence of the chemically crosslinked network (Figure S13, Supporting Information). Another example of elasticity for NBR/IBIL hybrid electrolyte is shown in Figure 3b, in which the hybrid electrolyte exhibits 86% strain recovery after 6 s and leaves only 0.6% strain after 15 min, highlighting the low hysteresis of this lithium‐conducting elastomer.

To gain deeper insight into the NBR/IBIL hybrid electrolyte, a modulus of resilience (*U*_r_) has been introduced to quantify its elasticity, which is the maximum reversible stored energy in the material and can be given by the area under the linear stress–strain curve. *U*_r_ of the NBR/IBIL hybrid electrolyte (0.92 MJ m^–3^) is ≈2.2 times that of PBA/SN electrolyte (0.41 MJ m^−3^),^[^ ^14^ ^]^ ≈2.9 times that of SiO_2_/PPO electrolyte^[^ ^15^ ^]^ (0.32 MJ m^−3^), and much higher than the values for other reported electrolytes^[^ ^10^, ^26^, ^27^, ^29^, ^30^ ^]^ (Figure 3c and Table S1, Supporting Information). To the best of our knowledge, the NBR/IBIL hybrid electrolyte is one of the toughest SPEs with relatively high ionic conductivity reported to date. The durability property of the NBR/IBIL hybrid electrolyte was also investigated. The elastic electrolyte can undergo up to 1000 cycles at a high strain of 50% with negligible hysteresis (Figure 3d and Figure S14, Supporting Information). Such long‐lasting durability is necessary in order to accommodate the repeated electrode‐level volume changes of metallic lithium anodes and ensure stable contact with the electrodes. So far, very few elastic electrolytes have achieved such long cycling at high strains^[^ ^15^ ^]^ (Figure 3e). NBR/IBIL hybrid electrolyte with high resilience modulus and long fatigue durability ensures its stable contact with the electrodes, which would lay a solid foundation for the stable electrochemical performance of long‐term solid‐state lithium metal batteries.

**Figure 4:**
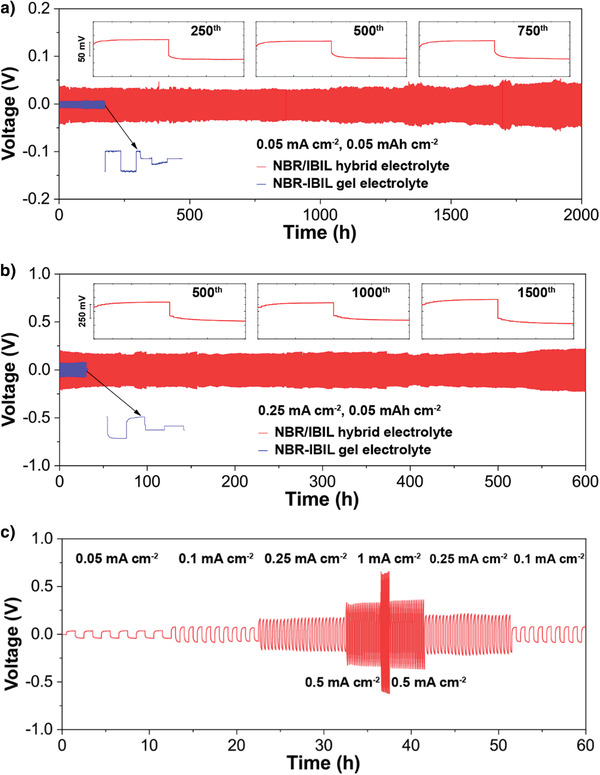
Electrochemical tests of Li|Li symmetrical batteries with NBR‐based electrolytes at 30 °C. Li|Li symmetrical battery cycling with NBR/IBIL hybrid and NBR‐IBIL gel electrolytes at current densities of a) 0.05 mA cm^–2^ for 0.05 mAh cm^–2^ and b) 0.25 mA cm^–2^ for 0.05 mAh cm^–2^. c) Rate performance of a solid‐state Li|Li symmetrical battery with the hybrid electrolyte at various current densities from 0.05 to 1 mA cm^–2^ for 0.05 mAh cm^–2^.

To evaluate the electrochemical stability of the NBR/IBIL hybrid electrolyte, solid‐state Li|Li symmetrical batteries with NBR‐based electrolytes were assembled and tested at 30 °C. As shown in **Figure** **4**a, the NBR/IBIL hybrid electrolyte reaches stable and extremely long stripping/plating cycling of 2000 h with a slight increase in impedance (Figure S15, Supporting Information), a performance which is superior to those of NBR and v‐NBR electrolytes (<0.1 h, Figure S16, Supporting Information). In contrast, although the NBR‐IBIL gel electrolyte with high ionic conductivity of ≈1.1 × 10^–3^ S cm^–1^ exhibits a much lower overpotential (≈6 mV, Figure S17, Supporting Information) than the NBR/IBIL hybrid electrolyte (≈34.5 mV), it displays a hard short circuit within 175 h due to the poor mechanical strength caused by the gelation of a large number of free IBIL molecules. The contrast in cycling life is even greater at a higher current density of 0.25 mA cm^–2^ for 0.05 mAh cm^–2^, where the cycle time of the gel electrolyte is only one‐twentieth that of the NBR/IBIL hybrid electrolyte (30 h, Figure S18, Supporting Information). After cycling for 2000 and 600 h at 0.05 and 0.25 mA cm^−2^, the metallic lithium anodes still exhibit smooth and compact surfaces (Figure S19, Supporting Information). The symmetrical battery with the hybrid electrolyte continues to operate well even when the current density increases to 1.0 mA cm^–2^ for 0.05 mAh cm^–2^, and no lithium dendrites can be observed on the surface and cross‐section of the metallic lithium anode after cycling (Figure 4c and Figures S20 and 21, Supporting Information). These results demonstrate that by virtue of its high resilience and fatigue durability, the NBR/IBIL hybrid electrolyte can provide stable interfacial contact and inhibit the growth of lithium dendrites during long‐term operation.

**Figure 5:**
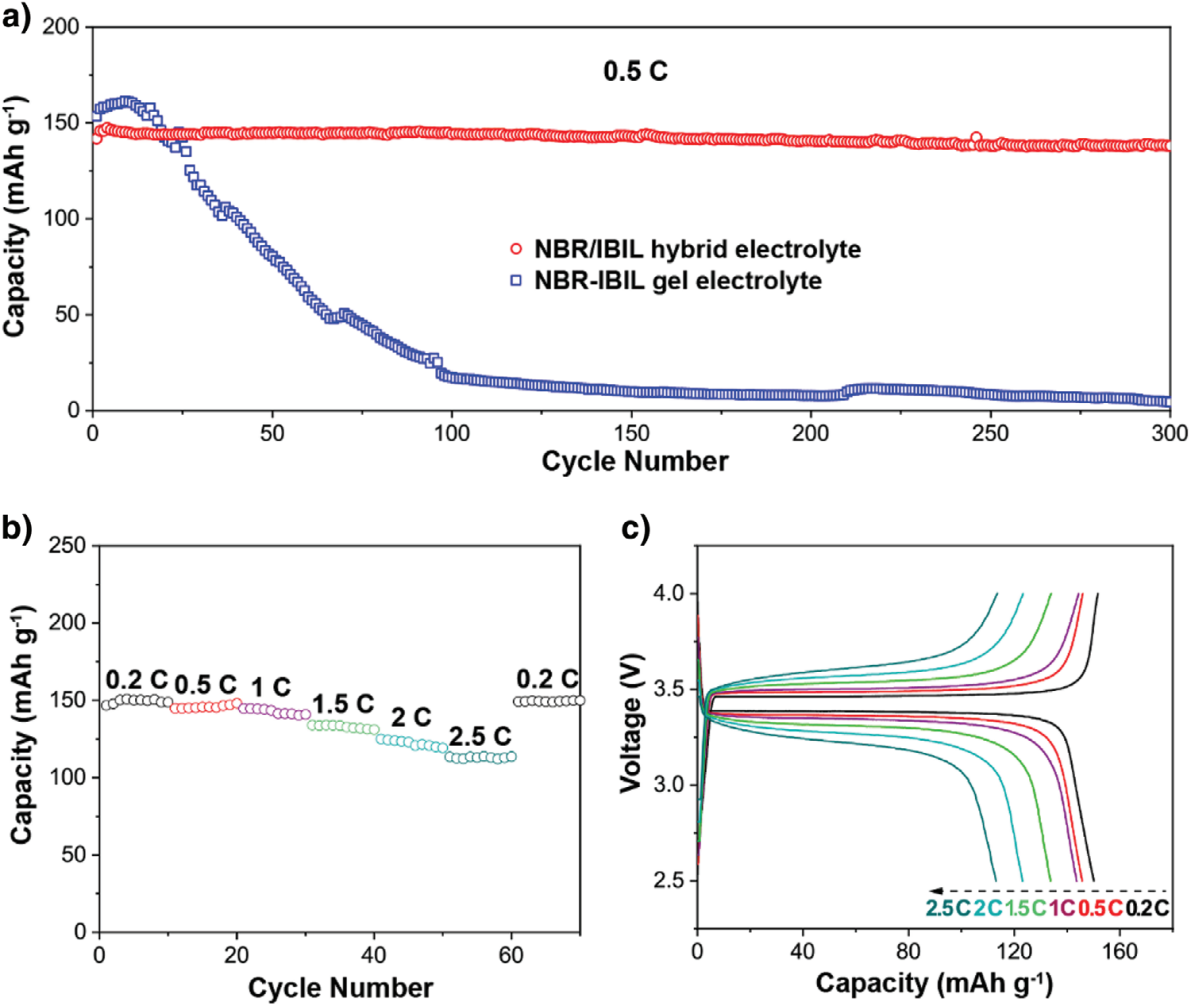
Electrochemical performance of Li/LFP batteries with NBR‐based electrolytes. a) Cycling stability of Li/LFP batteries with the NBR/IBIL hybrid electrolyte and an NBR‐IBIL gel electrolyte at 0.5 C under 30 °C. b) Rate capability of the hybrid electrolyte and c) its corresponding charge/discharge curves at different C rates.

The electrochemical performance of the NBR/IBIL hybrid electrolyte in full batteries was further evaluated using metallic lithium foil as the anode and a standard LiFePO_4_ (LFP) cathode. The full battery's cyclic voltammograms (CV) were recorded at 0.5 mV s^–1^, and typical characteristic redox peaks of Fe^2+^/Fe^3+^ couple for LFP cathodes^[^ ^31^ ^]^ were detected at ≈3.7/3.2 V (Figure S22, Supporting Information). The CV curves are highly reversible, suggesting that the hybrid electrolyte has good electrochemical stability. The full battery using our NBR/IBIL hybrid electrolyte delivers long‐term cycling stability with a good capacity retention of 94.3% and a small increase in impedance after 300 cycles at 0.5 C (**Figure** **5**a and Figures S23–S25, Supporting Information; C = 170 mA g^–1^). In contrast, full batteries using NBR, v‐NBR, and NBR‐IBIL gel electrolytes failed rapidly (Figure S26, Supporting Information and Figure 5a), the first two of which were attributed to extremely poor ionic conductivities and the last one to insufficient mechanical performance. Furthermore, owing to the high ionic conductivity of the NBR/IBIL hybrid electrolyte, the full battery also exhibits good rate capabilities (Figure 5b and Figure S27, Supporting Information). Even when the current density increases to 2.5 C, the capacity remains as high as 113 mAh g^–1^, and a well‐defined discharge voltage plateau^[^ ^32^ ^]^ can be observed (Figure 5c). When the current density decreases to 0.2 C, the observed capacity (149 mAh g^–1^) is comparable to the initial value, confirming the good cycling stability of the hybrid electrolyte. A Li/LFP battery with NBR/IBIL hybrid electrolyte and a high loading LFP cathode was also performed at 0.2 C (Figure S28, Supporting Information). The full battery achieves an areal capacity of ≈1.1 mAh cm^–2^, which is higher than that of a full battery with SiO_2_/PPO electrolyte at 0.2 C under 25 °C.^[^ ^15^ ^]^ The safety of solid‐state pouch cells using the NBR/IBIL hybrid electrolyte was further confirmed by abuse testing. The solid‐state pouch cell continues to power an LED at room temperature even when subjected to bending, puncturing, and cutting (Figure S29, Supporting Information), proving that the NBR/IBIL hybrid electrolyte can ensure safe and stable operation of solid‐state pouch cells.

## 3 Conclusion

In conclusion, a rubber‐derived lithium‐conducting elastomer has been fabricated using the vulcanization approach. By virtue of the chemically crosslinked network consisting of NBR chains and IBIL fragments generated during vulcanization, both mechanical performance and lithium‐ion transport of NBR/IBIL hybrid elastomer can be guaranteed. Hence, elastic NBR/IBIL hybrid electrolyte achieves high resilience of 0.92 MJ m^−3^, long‐lasting fatigue durability, and high room‐temperature ionic conductivity of 2.7 × 10^−4^ S cm^−1^ to enable intimate contact and fast lithium‐ion transport, resulting in stable, long‐cycling, and high‐performance solid‐state lithium metal batteries. Given the rich variety, diverse structures, and modifiable properties of rubber materials, we believe this vulcanization method can be extended to explore a range of lithium‐conducting elastomers with highly tunable mechanical properties and good ionic conductivity for practical applications in solid‐state lithium metal batteries.

## Conflict of Interest

The authors declare no conflict of interest.

## Supporting information

Supporting Information

## Data Availability

The data that support the findings of this study are available from the corresponding author upon reasonable request.
