# Supplementary material for: A Highly Durable Rubber‐Derived Lithium‐Conducting Elastomer for Lithium Metal Batteries
Source: Adv Sci (Weinh). 2022 Mar 31;9(16):2200553. doi: 10.1002/advs.202200553 (PMC9165490; doi:10.1002/advs.202200553)
Supplement: Supplementary file 1 — Supporting Information [file ADVS-9-2200553-s001.pdf]

ADVANCED SCIENCE
Open Access

Supporting Information

for *Adv. Sci.*, DOI 10.1002/advs.202200553

A Highly Durable Rubber-Derived Lithium-Conducting Elastomer for Lithium Metal Batteries

Yongzheng Shi, Na Yang, Jin Niu, Shubin Yang* and Feng Wang**

Supporting Information

A highly durable rubber-derived lithium-conducting elastomer for lithium metal batteries

Yongzheng Shi[1,2], Na Yang[1,2], Jin Niu[1,2,*], Shubin Yang[3,*] Feng Wang[1,2,*]

Dr. Y. Z. Shi, Dr. N. Yang, Prof. J. Niu, Prof. F. Wang
[1]State Key Laboratory of Chemical Resource Engineering, Beijing Key Laboratory of Electrochemical Process and Technology for Materials, Beijing University of Chemical Technology, Beijing 100029, P. R. China
[2]Beijing Advanced Innovation Center for Soft Matter Science and Engineering, Beijing University of Chemical Technology, Beijing 100029, P. R. China
*Email: niujin@mail.buct.edu.cn, wangf@mail.buct.edu.cn
Prof. S. B. Yang
[3]School of Materials Science and Engineering, Beihang University, 100191, Beijing, China
*Email: yangshubin@buaa.edu.cn

Experimental Section

Preparation of NBR/IBIL hybrid electrolytes: Elastic hybrid electrolyte was synthesized via sulfur vulcanization of nitrile butadiene rubber (NBR, Baymod N XL 33.61 from LANXESS with 33 percent acrylonitrile proportion) with polymerizable ionic liquid, which was named as NBR/IBIL hybrid electrolyte. Typically, sulfur powder, lithium bis(trifluorosulfonyl)imide (LiTFSI), and ionic liquid (1-allyl-3-vinylimidazolium bis(trifluorompropylsulfonyl)imide, abbreviated as IBIL) were first dissolved in acetone/xylene solution (V:V=2:1), then NBR powder was slowly added under stirring. The concentration of NBR was 0.1 g mL^{-1} accompanied by 4 wt.% of the vulcanizing agent, and the addition amounts of ionic liquid and lithium salt were 4.8 mmol g_{NBR}^{-1} of IBIL, 1.7 mmol g_{NBR}^{-1} of LiTFSI in NBR, respectively. The mixture was stirred at 50 °C for 36 h under 750 rpm to obtain a homogeneous slurry, and the slurry was uniformly coated on glass using a film applicator. After evaporating solvents for 2 h at room temperature and 12 h at 80 °C, NBR/IBIL hybrid electrolyte was obtained via

vulcanizing at 180 °C for 1 h under nitrogen atmosphere. It is noted that after evaporation of solvents, the slurry yields a gel electrolyte denoted NBR-IBIL gel electrolyte. For comparison, NBR and vulcanized NBR (denoted v-NBR) electrolytes were also prepared in the same procedures except that IBIL/sulfur agent or IBIL was not added to the precursor slurries, respectively. NBR, and NBR/IBIL hybrid membranes without LiTFSI were also fabricated to determine the changes in element valence before and after vulcanization.

Material Characterizations: The morphology and element distribution of NBR-based membranes were obtained from a field emission scanning electron microscope (FESEM, JSM-7500). X-ray diffraction (XRD) patterns were characterized on a Rigaku D/Max 2500 X-ray diffractometer with CuKα radiation. Fourier transform infrared spectroscopy (FTIR) spectra of NBR-based membranes were measured on a Nicolet Nexus 670 spectrometer in attenuated total reflection (ATR) mode. Differential scanning calorimetry (DSC) and thermogravimetric analysis (TGA) were carried out using DSC-Q20 and TGA-Q50 from TA Instruments at a ramp rate of 10 °C in air, respectively. Chemical compositions and elemental states of NBR-based membranes were investigated using Thermo Fisher Scientific ESCALAB 250 with Al Kα-radiation. Binding energy values of C1s peaks were calibrated to be 284.8 eV. Solid-state ^{7}Li NMR spectra of electrolyte membranes were measured in a double resonance 3.2 mm magic angle spinning probe on a JNM-ECZ600R spectrometer with a spinning frequency of 12 kHz and a single pulse sequence, and the ^{7}Li shifts were referenced to LiCl (-1.19 ppm). Tensile strength and creep tests were performed on DMA Q800 (TA Instruments) at a strain rate of 100% min^{-1} and step stress of 0.1 MPa at 25 °C, respectively.

Electrochemical Measurements: Ionic conductivities of elastic electrolytes were calculated

using the following equation:

$$\sigma = d/(R_b \times A) \tag{1}$$

where d was the thickness, R_b was the bulk resistance, and A was the area of elastic electrolytes. R_b of elastic NBR-based electrolytes was determined at room temperature by assembling electrolytes discs (Φ, ~16 mm; thickness, ~120 μm) in 2032 coin cells sandwiched between two steel spacers and using electrochemical impedance spectroscopy (EIS) on a CHI 760E electrochemical workstation (Chenhua, Shanghai) in the frequency range of 1 MHz to 10 mHz with an amplitude of 5 mV. Li ion transference numbers (t_{Li^+}) of NBR/IBIL hybrid and NBR-IBIL gel electrolytes were measured at 25 °C using potentiostatic polarization method proposed by Bruce and Appetecchi [1-2]. After applying a polarization potential of 10 mV (ΔV), t_{Li^+} in the symmetrical battery system can be calculated using the following equation:

$$t_{\mathrm{Li}^+} = \frac{I_{\mathrm{ss}}}{I_0} \times \frac{\Delta V - I_0 R_0}{\Delta V - I_{\mathrm{ss}} R_{\mathrm{ss}}} \tag{2}$$

where I_0 is the initial current, I_{ss} is the steady-state current, and R_0 and R_{ss} are the corresponding initial and steady state resistances, respectively.

Cyclic voltammetry (CV) measurement of the NBR/IBIL hybrid electrolyte was conducted on the CHI 760E electrochemical workstation in the voltage range of -0.7 V to 6 V at 1 mV s^{-1} in 2032 coin cells with Li|NBR/IBIL hybrid electrolyte|steel spacers configuration. Linear sweep voltammetry (LSV) of NBR and v-NBR electrolytes were carried out from 2.5 V to 5 V at 1 mV s^{-1}. The cells were assembled in an Ar-filled glovebox (<0.1 ppm H_2O and O_2). Li|NBR-based electrolyte|Li and Li|NBR-based electrolyte|$LiFePO_4$ cells were assembled to evaluate the electrochemical performance of NBR-based electrolytes. Standard $LiFePO_4$ cathodes contained 80 wt.% $LiFePO_4$, 10 wt.% poly(vinylidene fluoride), and 10 wt.% carbon black with

typical active material loading larger than 1 mg cm^{-2}. Interfacial resistances were alleviated by wetting 10 μL of liquid electrolyte (1 M LiTFSI in 1,3-dioxolane/1,2 dimethoxyethane (V:V=1:1) with 1 wt.% lithium nitrate) between electrolytes and electrodes.

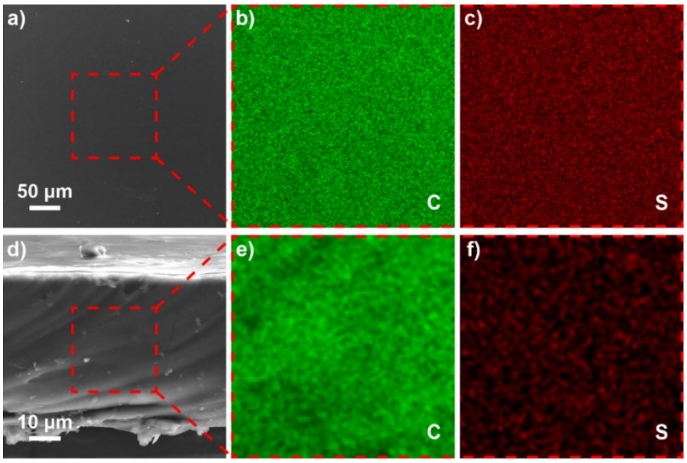

Figure S1. SEM images of (a) surface and (d) cross section for v-NBR membrane, and their corresponding energy dispersive X-ray spectroscopy (EDS) mapping images (b-c) and (e-f), respectively. The EDS mapping images reveal that the sulfur vulcanizing agent is uniformly distributed on the surface and inside the v-NBR membrane.

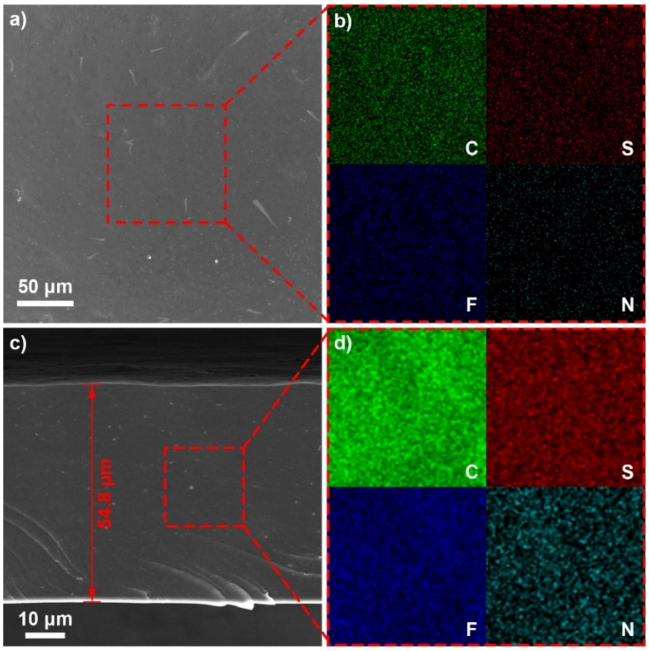

Figure S2. SEM images of (a) surface and (c) cross section for NBR/IBIL hybrid electrolyte, and their corresponding EDS mapping images (b) and (d), respectively. The NBR/IBIL hybrid electrolyte membrane is as thin as 54.8 μm, and the corresponding EDS images reveal that IBIL and LiTFSI are homogeneously distributed on the surface and inside the NBR/IBIL hybrid membrane.

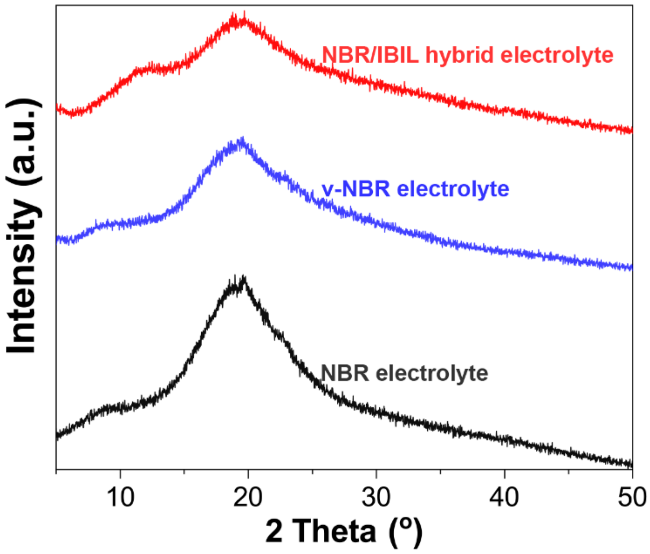

Figure S3. XRD patterns of NBR/IBIL hybrid, v-NBR, and NBR electrolytes. No clear diffraction peaks of crystal LiTFSI can be observed in the XRD patterns of NBR/IBIL hybrid electrolyte, implying that lithium salt is uniformly distributed in the hybrid electrolyte[3-4]. Furthermore, NBR/IBIL hybrid electrolytes exhibit XRD patterns similar to those of NBR electrolytes, but the intensity is less than two-thirds that of the NBR electrolyte under the same test conditions, revealing that grafting IBIL fragments may increase the amorphous regions of NBR/IBIL hybrid electrolyte, thus facilitating local segmental motions of polymer.

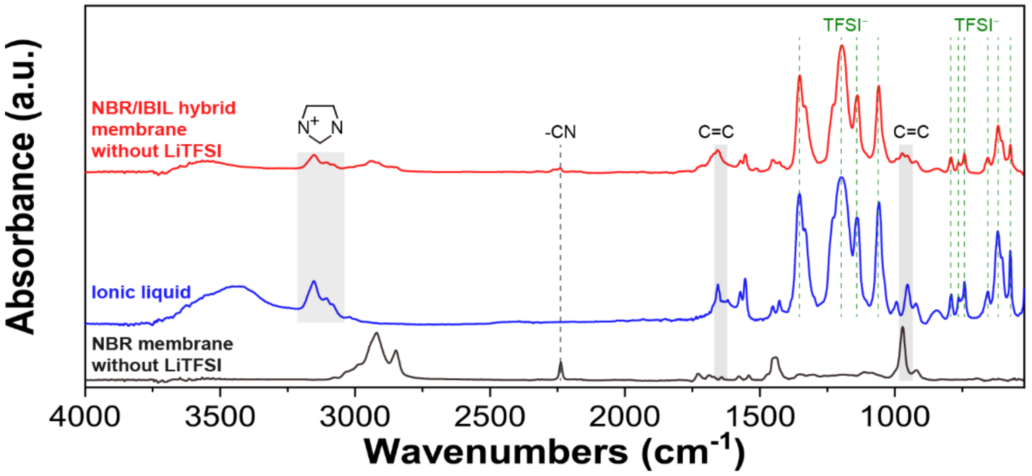

Figure S4. FTIR spectra of NBR membrane, IBIL, and NBR/IBIL hybrid membrane. Typical functional groups of NBR and IBIL can be found in the FTIR spectrum of the NBR/IBIL hybrid membrane. For instance, stretching vibration of nitrile for NBR locates at 2238 cm^{-1}. Furthermore, stretching vibration region of -NH in imidazolium ring locates at 3151 cm^{-1}. And characteristic peaks of $TFSI^-$ anion centered at 571, 616, 654, 741, 763, 791, 1059, 1137, 1195, and 1351 cm^{-1} can be observed in the FTIR spectrum of NBR/IBIL hybrid. These results demonstrate that NBR/IBIL hybrid matrix has been successfully fabricated. Moreover, the -C=C- peak intensity of the hybrid matrix centered at 970 cm^{-1} is significantly lower than that of NBR and IBIL, demonstrating that large consumption of -C=C- bonds occurred during vulcanization. Noted that a broad water peak can be observed at 3562 cm^{-1} because the electrolyte was exposed to air for a long time before the test, which would be avoided by quickly transferring to the glove box after sulfur vulcanization.

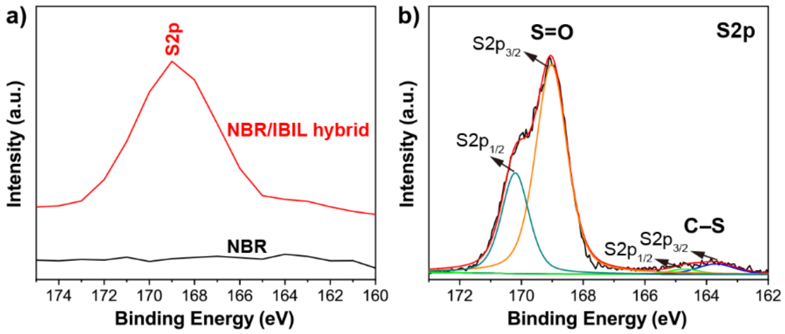

Figure S5. (a) XPS survey spectra of NBR and NBR/IBIL hybrid matrix. (b) S2p spectra of NBR/IBIL hybrid matrix. S2p peaks can be observed in the XPS survey spectrum of NBR/IBIL hybrid. Furthermore, S2p spectra of the hybrid matrix demonstrate the presence of -C-S- bond, which indicates the formation of the chemically crosslinked network during vulcanization.

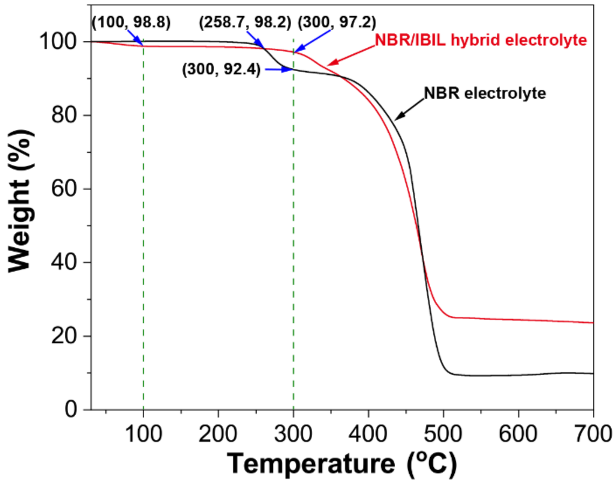

Figure S6. TGA curves of NBR and NBR/IBIL hybrid electrolytes. NBR/IBIL hybrid electrolyte suffers only ~1.8 wt.% mass drop from 30 to 250 ºC, demonstrating that only a few small molecules are free in the electrolyte. What's more, even when the temperature rises to 300 ºC, NBR/IBIL hybrid electrolyte suffers only ~2.8 wt.% mass drop, which is about one-third of that for NBR electrolyte (~7.6 wt.%), further proving that the vulcanization approach is effective in enhancing the thermal stability of NBR-based elastomers.

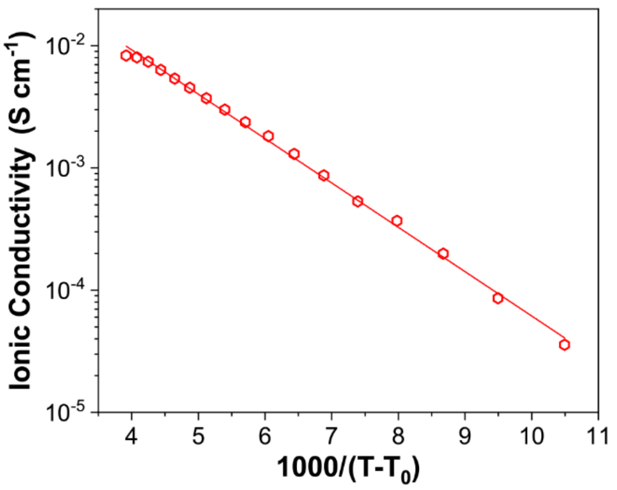

Figure S7. VTF fitting of temperature-dependent ionic conductivities for the NBR/IBIL hybrid electrolyte. T_0 is the glass transition temperature of the hybrid electrolyte. The temperature-dependent ionic conductivities of the NBR/IBIL hybrid electrolyte are well-fitted by the Vogel-Tammann-Fulcher equation, indicating its lithium-ion transport behavior as solid-state electrolytes.

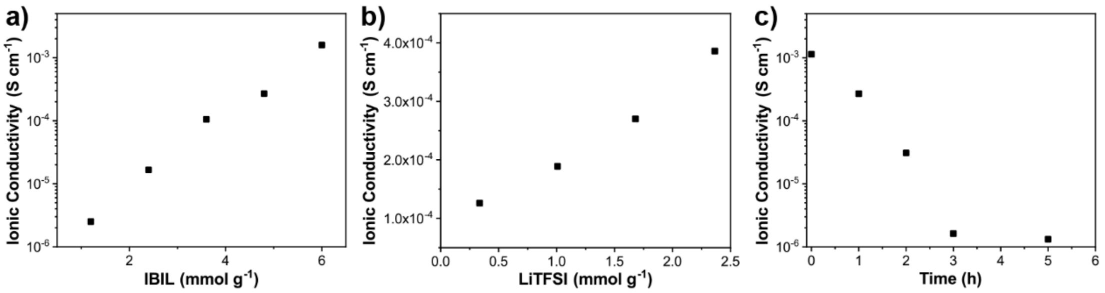

Figure S8. Ionic conductivities of NBR/IBIL hybrid electrolytes measured at room temperature with increasing (a) IBIL and (b) LiTFSI contents and (c) curing time. As shown in Figure S1-3, the conductivities of NBR/IBIL hybrid electrolyte increase with increasing IBIL and LiTFSI levels and decreasing curing time. But high-levels of IBIL or LiTFSI and short curing time will weaken the mechanical strength of the NBR/IBIL hybrid electrolyte. As a result, we chose 4.8 mmol g^{-1} of IBIL, 1.7 mmol g^{-1} of LiTFSI, and 1 h of curing time to fabricate the elastic NBR/IBIL hybrid electrolyte.

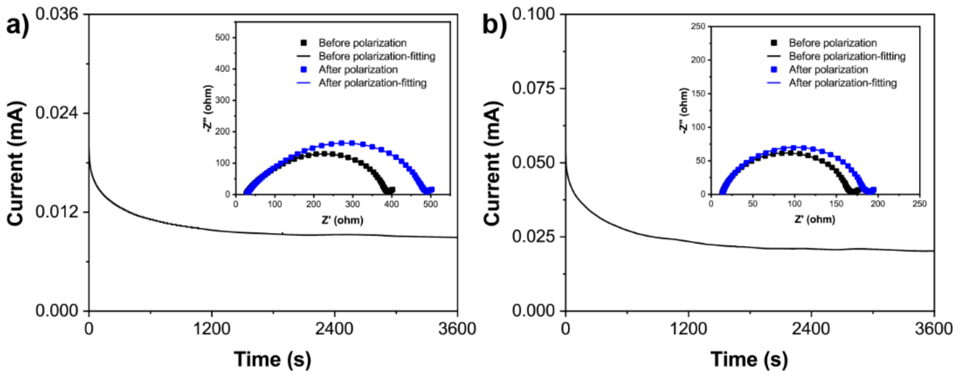

Figure S9. Li-ion transference number measurements of (a) NBR/IBIL hybrid electrolyte and (b) NBR-IBIL gel electrolyte. NBR/IBIL hybrid and NBR-IBIL gel electrolytes have low Li-ion transference numbers of 0.22 and 0.1, respectively, which can be attributed to the non-immobilization of the anion.

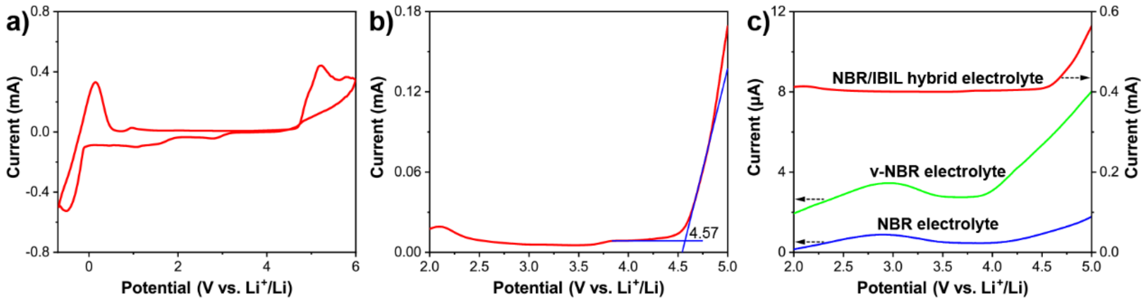

Figure S10. (a) CV curves of NBR/IBIL hybrid electrolyte with (b) corresponding magnified curve at high potential. (c) LSV curves of NBR, v-NBR, and NBR/IBIL hybrid electrolytes. The NBR/IBIL hybrid electrolyte shows the widest electrochemically stable window among NBR-based electrolytes, demonstrating that IBIL fragments enhance the electrochemical stability of the NBR/IBIL hybrid electrolyte.

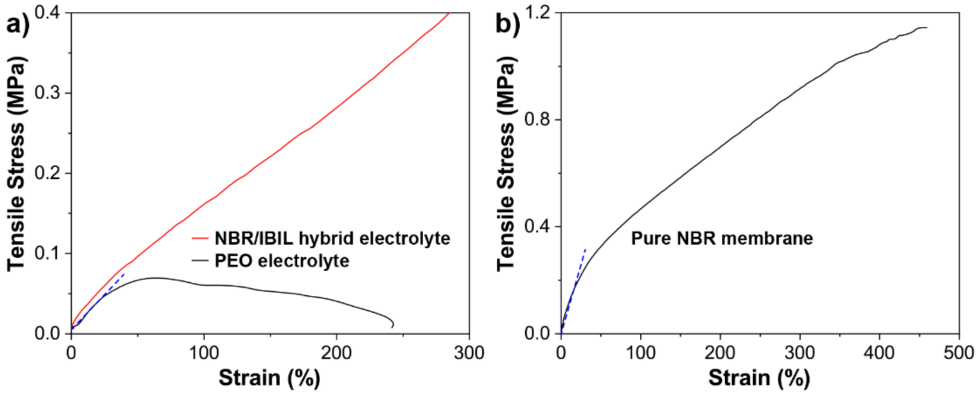

Figure S11. Stress-strain curves of (a) NBR/IBIL hybrid and PEO electrolytes and (b) pure NBR membrane. The NBR/IBIL hybrid electrolyte presents a linear stress-strain curve similar to v-NBR, but PEO electrolyte and pure NBR membrane undergo nonlinear stress-strain at strains greater than 30%, indicating the high resilience of the NBR/IBIL hybrid.

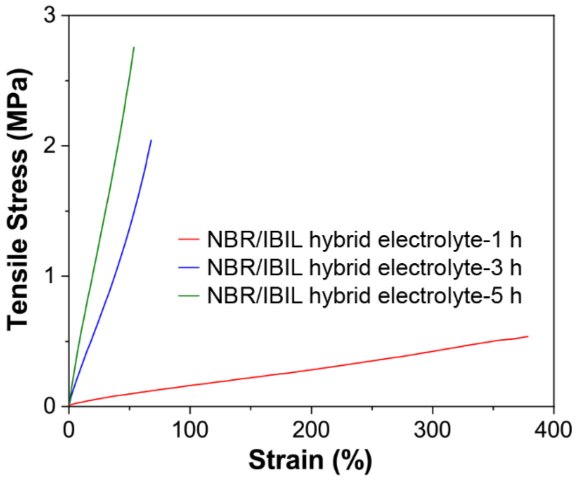

Figure S12. Stress-strain curves of NBR/IBIL hybrid electrolytes tested at room temperature with increasing curing time. All the NBR/IBIL hybrid electrolytes display linear stress-strain curves similar to v-NBR due to the chemically crosslinked network. As the curing time increases, the tensile stresses of hybrid electrolytes at break increase rapidly, which are higher than that of v-NBR electrolyte.

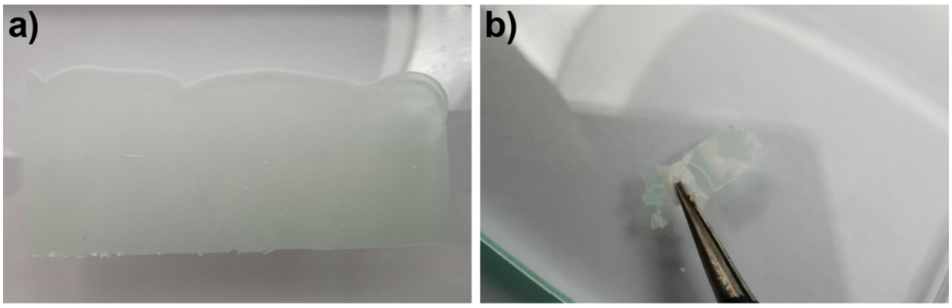

Figure S13. (a-b) Digital pictures of NBR-IBIL gel electrolyte. This gel electrolyte loses the elasticity of NBR and tends to fracture after small deformations, which reveals that IBIL would significantly destroy the mechanical strength of NBR-based electrolytes due to the absence of the sulfur vulcanization process.

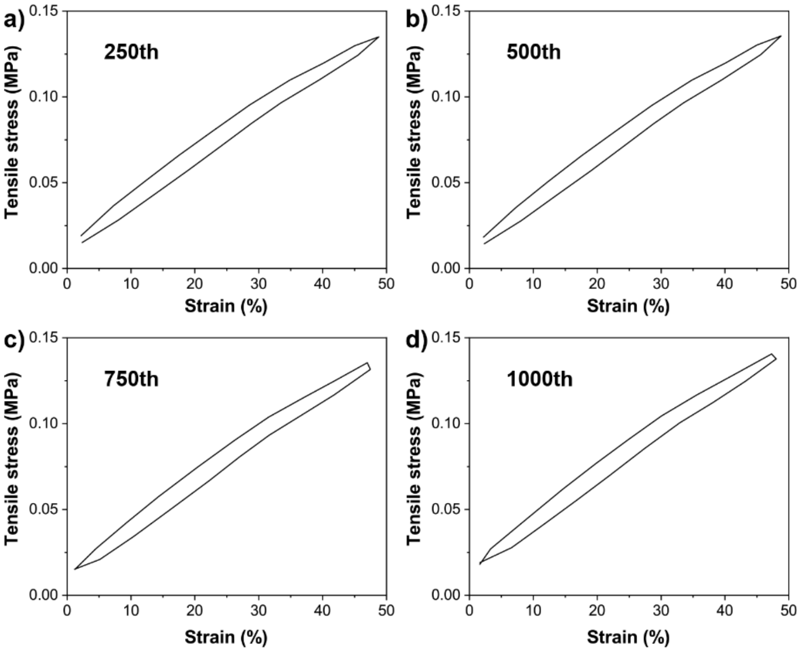

Figure S14. (a-d) Strain-stress curves of NBR/IBIL hybrid electrolyte at 50% strain for various cycles. The electrolyte has a low hysteresis at the initial cycles, which remain at low levels even after 1000 cycles.

Modulus of resilience (U_r) is the maximum reversible stored energy in the material and can be described as an area under the linear stress-strain curve. It can be calculated by the following equation:

$$U_r = \frac{\sigma_y^2}{2E} = \frac{\sigma_y \varepsilon_y}{2}$$

where E is the Young's modulus, σ_y is the yield strength, and ε_y is the yield strain. If the yield point cannot be clearly observed, stress-strain curve is approximated as linear; thus, tensile strength and elongation at break are used as σ_y and ε_y to calculate U_r from reference, respectively.

Table S1. Conductivities and modulus of resilience data in the comparison plot.

Reference	Lithium salt	Plasticizer	σ at RT [S cm^{-1}]	Yield Stress [MPa]	Resilience [MJ m^{-3}]	Reference
NBR/IBIL hybrid	15 wt.% LiTFSI	56 wt.% IBIL	2.7×10^{-4}	0.51	0.92	This work
v-NBR	33 wt. % LiTFSI	None	3.7×10^{-8}	1.29	0.86	This work
NBR	33 wt. % LiTFSI	None	9.8×10^{-8}	0.31	0.025	This work
poly-butyl acrylate/SN[a)]	22 wt. % LiTFSI	38.8 wt.% SN	1.1×10^{-3}	0.28	0.41	[5]
SiO_2/PPO	27 wt. % LiTFSI	30 wt.% PC	2.5×10^{-4}	0.45	0.32	[6]
PEO	40 wt. % LiTFSI	None	2.0×10^{-5}	0.04	0.003	[6]
Glass fiber/PEO	32 wt. % LiTFSI	10 wt.% fluoroethylene carbonate	3.0×10^{-5}	0.61	0.15	[7]
SiO_2/PEO	26 wt. % LiTFSI	None	5.4×10^{-6}	0.32	0.0048	[8]
Aramid fiber/PEO	Not reported	None	5.0×10^{-6}	61	0.38	[9]
PEGDA[b)]	30 wt. % LiTFSI	42 wt.% SN	8.0×10^{-4}	0.062	0.0015	[10]
PEGDA	30 wt. % LiTFSI	49 wt.% SN	1.0×10^{-3}	0.095	0.001	[10]
Celgard 3501	1 M $LiPF_6$	EC:DMC (1:1)	6.2×10^{-4}	5.5	0.067	[11]

[a)] SN is abbreviated from succinonitrile; [b)] PEGDA is abbreviated from poly(ethylene glycol) diacrylate

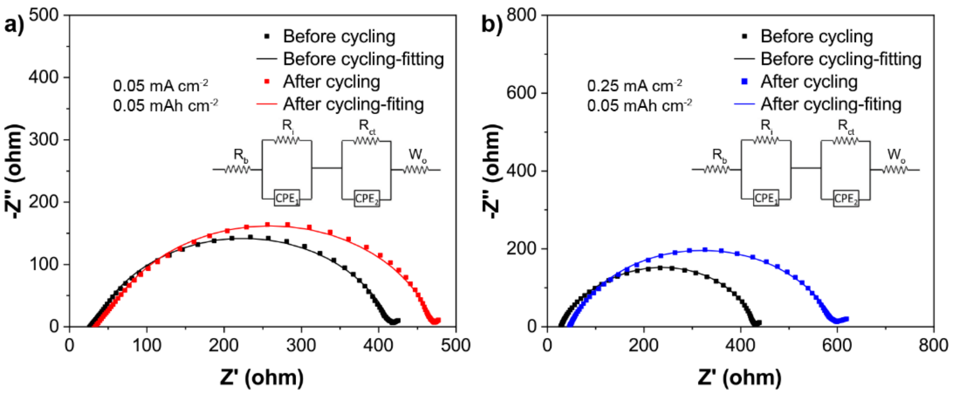

Figure S15. EIS plots of Li|Li symmetrical batteries with the NBR/IBIL hybrid electrolyte before and after cycling at (a) 0.05 mA cm^{-2} for 0.05 mAh cm^{-2} and (b) 0.25 mA cm^{-2} for 0.05 mAh cm^{-2} with an equivalent circuit in the inset. After cycling, the impedance of both batteries increases.

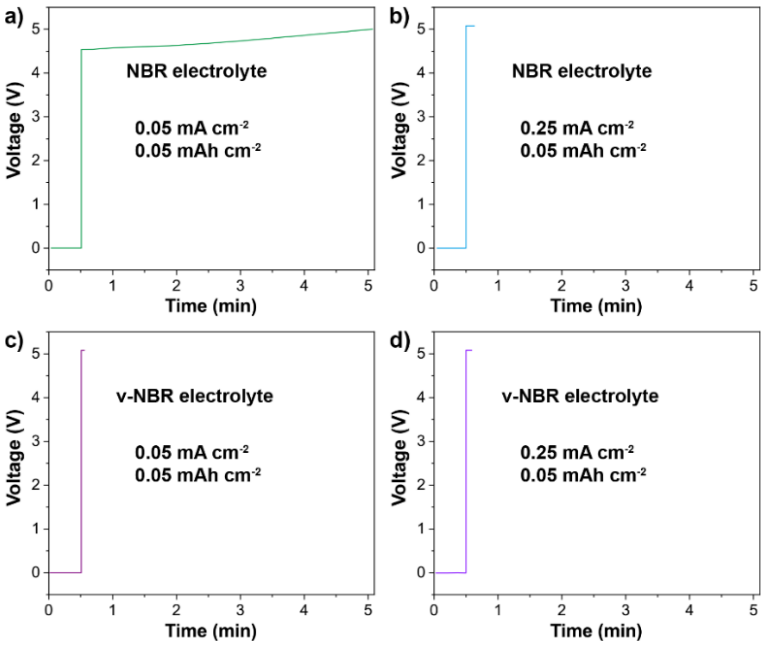

Figure S16. Time-voltage profile of lithium symmetrical batteries with (a-b) NBR and (c-d) v-NBR electrolytes at different current densities. Li|NBR|Li and Li|v-NBR|Li symmetrical batteries quickly failed (<0.1 h) at 0.05 and 0.25 mA cm^{-2} due to their poor ionic conductivities.

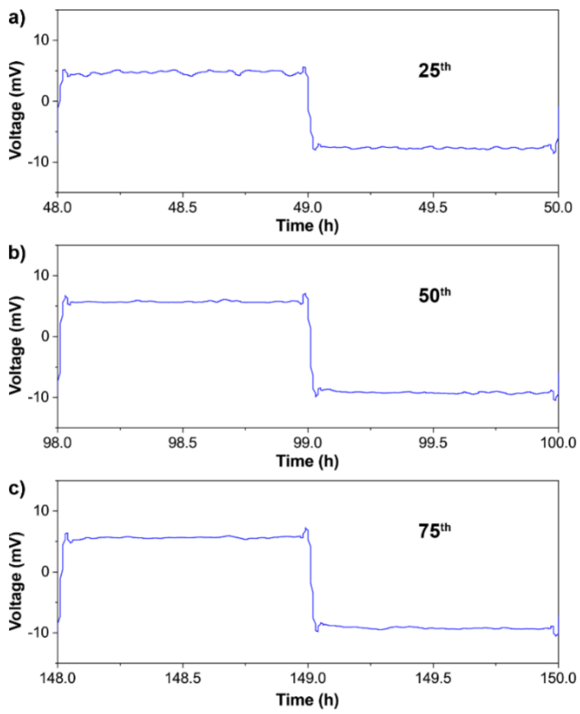

Figure S17. Time-voltage profile of Li|NBR-IBIL gel electrolyte|Li symmetrical batteries at 0.05 mA cm^{-2} for different times. The symmetrical batteries exhibit low overpotentials of ~6 mV due to the high ionic conductivity of the NBR-IBIL gel electrolyte.

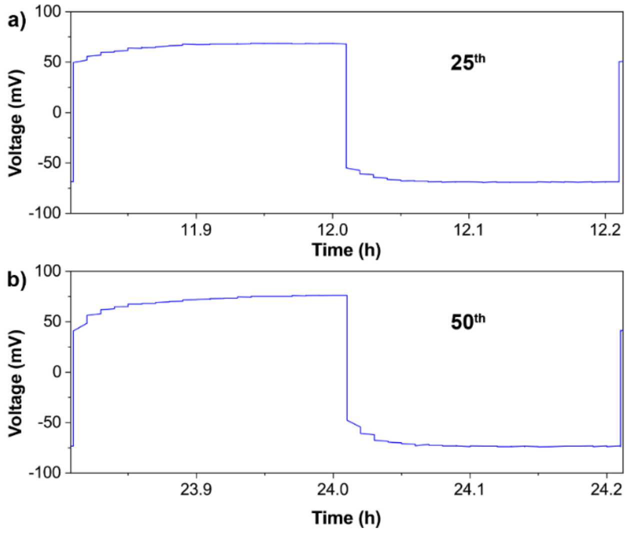

Figure S18. (a-b) Time-voltage profile of Li|NBR-IBIL gel electrolyte|Li symmetrical batteries at 0.25 mA cm^{-2} for different times. Owing to the high ionic conductivity of the NBR-IBIL gel electrolyte, the symmetrical battery exhibits low overpotentials of 76.6 mV.

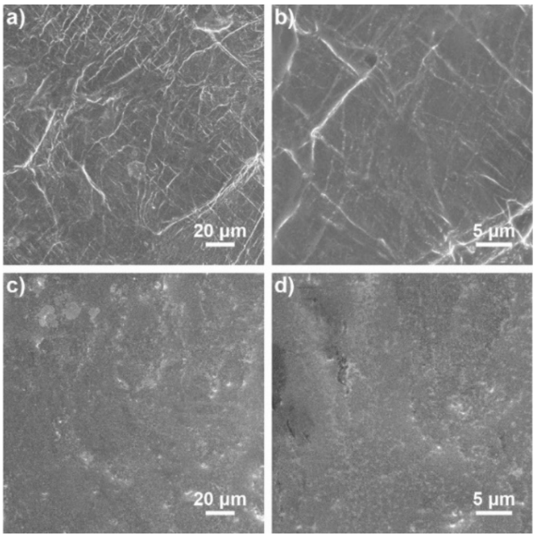

Figure S19. SEM images of metallic lithium anodes after cycling (a-b) at 0.05 mA cm^{-2} for 2000 h and (c-d) at 0.25 mA cm^{-2} for 600 h under 30 °C. Metallic lithium anodes exhibit smooth and compact surfaces after long cycling, revealing the ability of NBR/IBIL hybrid electrolyte to inhibit lithium dendrites.

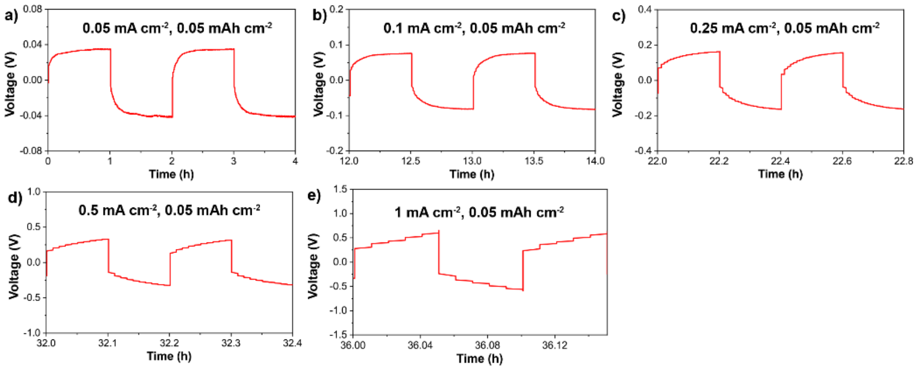

Figure S20. Time-voltage profile of Li|Li symmetrical batteries using NBR/IBIL hybrid electrolyte at different times with various current rates for 0.05 mAh cm^{-2}, which are (a) 0.05 mA cm^{-2}, (b) 0.1 mA cm^{-2}, (c) 0.25 mA cm^{-2}, (d) 0.5 mA cm^{-2}, and (e) 1 mA cm^{-2}. The corresponding overpotentials to the current rates are 35.2, 76.6, 161.4, 327.6, and 611.4 mV, respectively. Owing to the high ionic conductivity of the NBR/IBIL hybrid electrolyte, the corresponding symmetrical battery can operate well even when the current rate increases to 1.0 mA cm^{-2}.

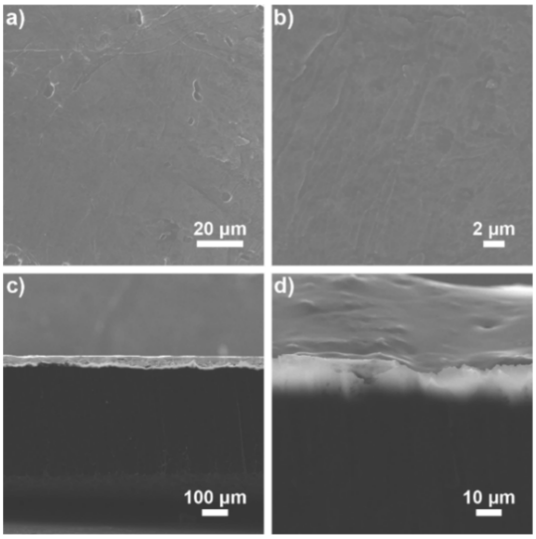

Figure S21. SEM images of (a-b) surface and (c-d) cross-section for metallic lithium anode after cycling at various current densities from 0.05 to 1 mA cm^{-2} for 0.05 mAh cm^{-2} under 30 °C. No lithium dendrites can be observed on the surface and cross-section of the metallic lithium anode after cycling at a high current density of 1 mA cm^{-2}, revealing the ability of NBR/IBIL hybrid electrolyte to inhibit lithium dendrites.

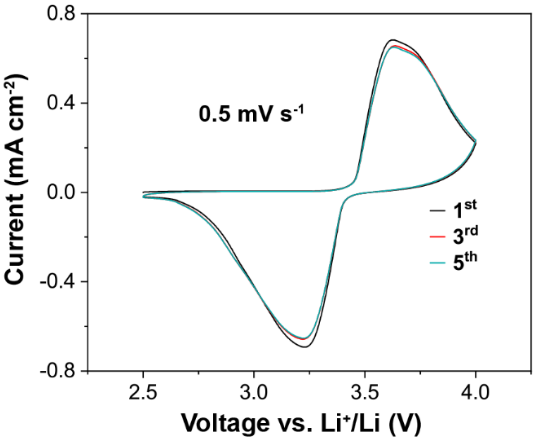

Figure S22. CV of Li/LFP battery with NBR/IBIL hybrid electrolyte at a scan rate of 0.5 mV s^{-1} under 30 °C. A pair of characteristic redox peaks for LFP cathodes can be observed at 3.7/3.2 V. These CV curves are almost overlapped, indicating that the solid-state battery is highly reversible and no significant side reactions are present, i.e., the NBR/IBIL hybrid electrolyte has good electrochemical stability.

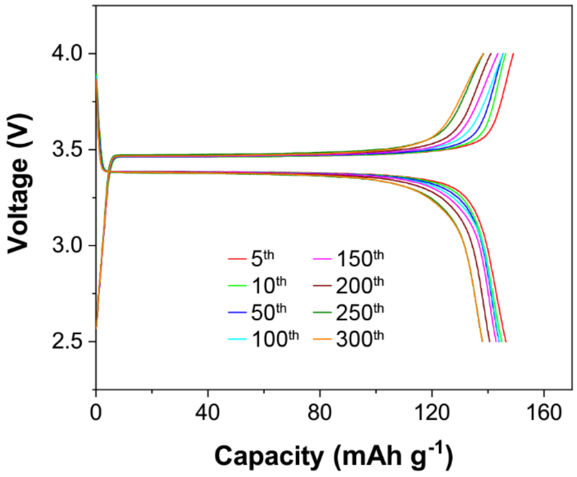

Figure S23. Charge/discharge curves of Li/LFP battery from 5th to 300th cycle with NBR/IBIL hybrid electrolyte at 0.5 C. Only a small increase in overpotentials can be observed from the 5th cycle to the 50th cycle, indicating that the solid-state Li/LFP battery with our NBR/IBIL hybrid electrolyte has long cycling stability.

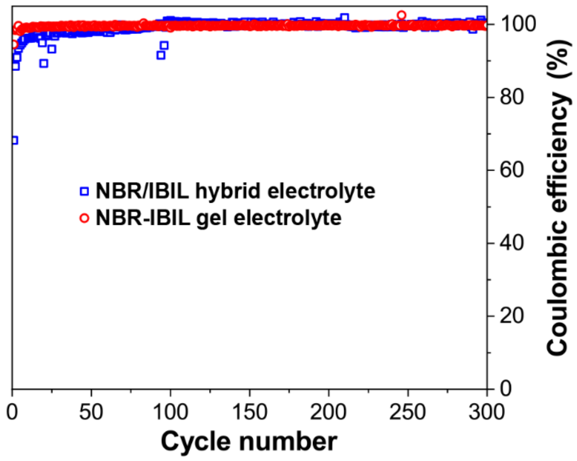

Figure S24. Coulombic efficiency of Li/LFP batteries with NBR/IBIL hybrid and NBR-IBIL gel electrolytes at 0.5 C under 30 ºC. The solid-state Li/LFP battery with NBR/IBIL hybrid electrolyte exhibits stable Coulombic efficiency after the first five cycles. In contrast, the Li/LFP battery with NBR-IBIL gel electrolyte suffers from unstable Coulombic efficiency due to its poor mechanical performance.

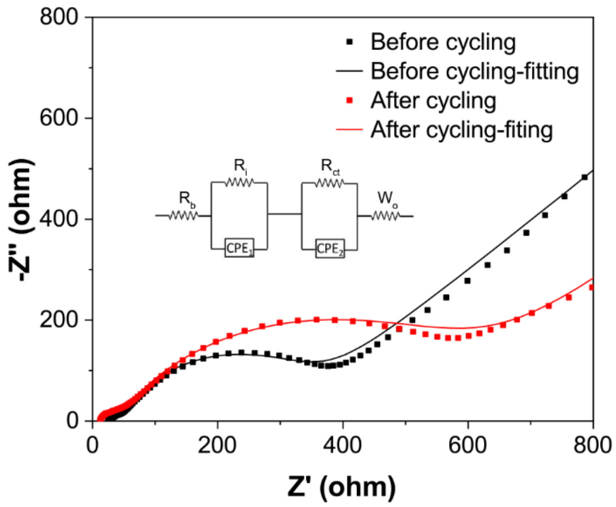

Figure S25. EIS plot of Li/LFP battery with NBR/IBIL hybrid electrolyte before and after cycling at 0.5 C with an equivalent circuit in the inset. The impedance of the Li/LFP battery shows a small increase after cycling.

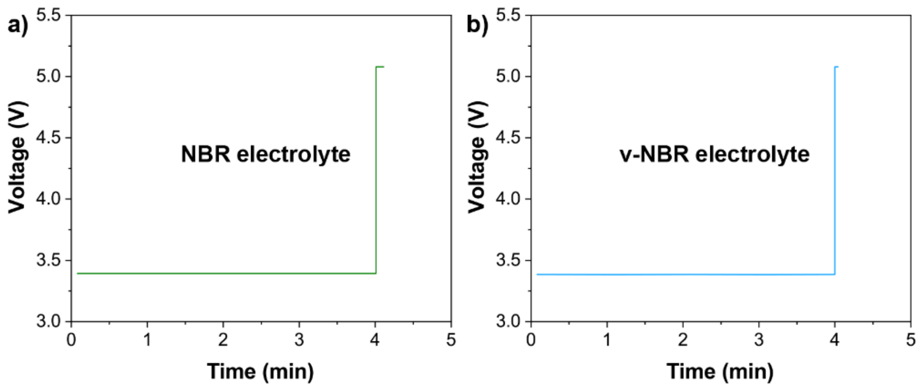

Figure S26. Galvanostatic discharge/charge profiles of Li/LFP batteries with (a) NBR and (b) v-NBR electrolytes at 0.5 C under 30 °C. As NBR and v-NBR electrolytes have poor ionic conductivities, their solid-state Li/LFP batteries can barely operate at 0.5 C and fail in less than 10 minutes.

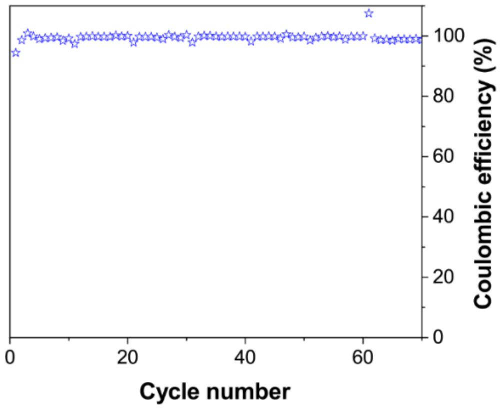

Figure S27. Coulombic efficiency of Li/LFP battery with NBR/IBIL hybrid electrolyte at various rates from 0.2 to 2.5 C. Owing to the high ionic conductivity of NBR/IBIL hybrid electrolyte, the solid-state full battery presents a stable Coulombic efficiency at various rates.

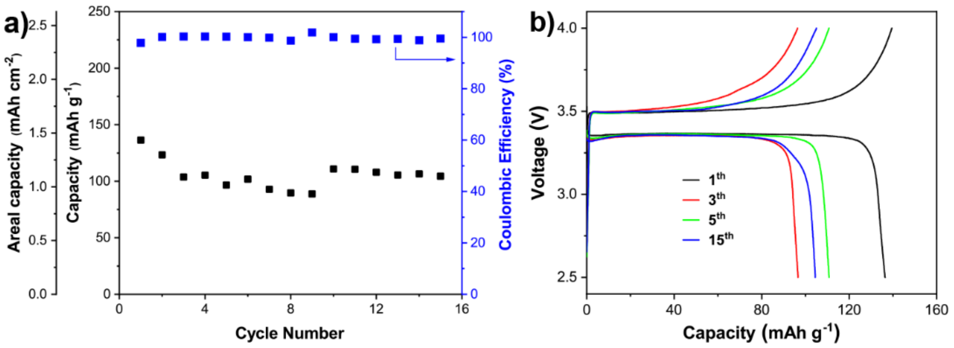

Figure S28. Electrochemical performance of a Li/LFP battery with NBR/IBIL hybrid electrolyte and a high loading LFP cathode (~10.5 mg cm^{-2}). (a) Cycling performance and (b) charge/discharge curves of the Li/LFP battery at 0.2 C under 30 ºC. The capacity and cycling stability of the Li/LFP battery with the high-loading LFP cathode are lower than those of the Li/LFP battery with a low-loading LFP cathode, which can be attributed to more severe interface problems caused by thick electrodes.

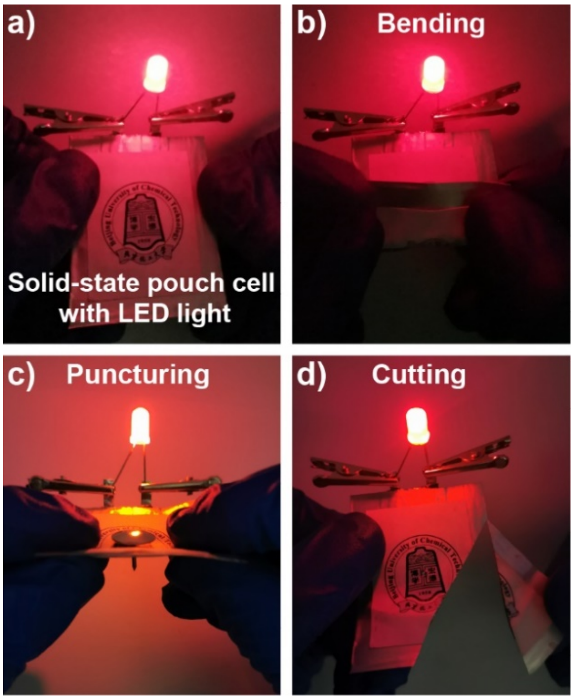

Figure S29. (a) Digital picture of solid-state pouch cell with a LED light lit at room temperature. (b-d) Digital pictures of the solid-state pouch cell connected to the LED being bent, punctured, and cut. The solid-state pouch cell assembled from metallic lithium anode, NBR/IBIL hybrid electrolyte, and standard LFP cathode operates well at ambient temperature, even when the pouch cell is bent, punctured, and cut, proving that NBR/IBIL hybrid electrolyte can ensure the safety and stability of its pouch cells during operating.

Supplementary References

[1] P. G. Bruce, J. Evans and C. A. Vincent, *Solid State Ionics*, **1988**, 28, 918–922.

[2] G. B. Appetecchi, G. Dautzenberg and B. Scrosati, *J. Electrochem. Soc.*, **1996**, 143, 6–12.

[3] C. Tao, M. H. Gao, B. H. Yin, B. Li, Y. P. Huang, G. Xu, J. J. Bao, *Electrochim. Acta* **2017**, 257, 31.

[4] T. Patodia, K. Sharma, S. Dixit, S. Katyayan, G. Agarwal, A. Jain, S. Jain, B. Tripathi, *Mater. Today: Proceedings* **2021**, 42, 1638.

[5] M. J. Lee, J. Han, K. Lee, Y. J. Lee, B. G. Kim, K.-N. Jung, B. J. Kim, S. W. Lee, *Nature* **2022**, 601, 217.

[6] J. Lopez, Y. Sun, D. G. Mackanic, M. Lee, A. M. Foudeh, M. S. Song, Y. Cui, Z. Bao, *Adv. Mater.* **2018**, 30, 1804142.

[7] G. Yang, M. L. Lehmann, S. Zhao, B. Li, S. Ge, P.-F. Cao, F. M. Delnick, A. P. Sokolov, T. Saito, J. Nanda, *Energy Storage Mater.* **2021**, 35, 431.

[8] S. Chen, Y. Zhao, J. Yang, L. Yao, X. Xu, *Ionics* **2017**, 23, 2603.

[9] S.-O. Tung, S. Ho, M. Yang, R. Zhang, N. A. Kotov, *Nat. Commun.* **2015**, 6, 1.

[10] M. Echeverri, C. Hamad, T. Kyu, *Solid State Ionics* **2014**, 254, 92.

[11] J. Cannarella, X. Liu, C. Z. Leng, P. D. Sinko, G. Y. Gor, C. B. Arnold, *J. Electrochem. Soc.* **2014**, 161, F3117.
